# Use of adenine base editing and homology-independent targeted integration strategies to correct the cystic fibrosis causing variant, W1282X

**DOI:** 10.1093/hmg/ddad143

**Published:** 2023-08-31

**Authors:** Karen Mention, Kader Cavusoglu-Doran, Anya T Joynt, Lúcia Santos, David Sanz, Alice C Eastman, Christian Merlo, Elinor Langfelder-Schwind, Martina F Scallan, Carlos M Farinha, Garry R Cutting, Neeraj Sharma, Patrick T Harrison

**Affiliations:** Department of Physiology, University College Cork, College Road, Cork, T12 K8AF, Ireland; School of Microbiology, University College Cork, College Road, Cork, T12 K8AF, Ireland; Department of Physiology, University College Cork, College Road, Cork, T12 K8AF, Ireland; McKusick-Nathans Department of Genetic Medicine, Johns Hopkins University, 3400 N. Charles Street, Baltimore, MD 21218, United States; Department of Physiology, University College Cork, College Road, Cork, T12 K8AF, Ireland; Faculty of Sciences, BioISI - Biosystems & Integrative Sciences Institute, University of Lisboa, Campo Grande, C8 bdg, Lisboa 1749-016, Portugal; Department of Physiology, University College Cork, College Road, Cork, T12 K8AF, Ireland; McKusick-Nathans Department of Genetic Medicine, Johns Hopkins University, 3400 N. Charles Street, Baltimore, MD 21218, United States; Division of Pulmonary and Critical Care Medicine, Department of Medicine, Johns Hopkins Hospital, 1800 Orleans St, Baltimore, MD 21287, United States; The Cystic Fibrosis Center, Lenox Hill Hospital, 100 E. 77th Street, 4E, New York, NY 10075, United States; School of Microbiology, University College Cork, College Road, Cork, T12 K8AF, Ireland; Faculty of Sciences, BioISI - Biosystems & Integrative Sciences Institute, University of Lisboa, Campo Grande, C8 bdg, Lisboa 1749-016, Portugal; McKusick-Nathans Department of Genetic Medicine, Johns Hopkins University, 3400 N. Charles Street, Baltimore, MD 21218, United States; McKusick-Nathans Department of Genetic Medicine, Johns Hopkins University, 3400 N. Charles Street, Baltimore, MD 21218, United States; Department of Physiology, University College Cork, College Road, Cork, T12 K8AF, Ireland

**Keywords:** CRISPR, adenine base editing, HITI, CFTR, W1282X

## Abstract

Small molecule drugs known as modulators can treat ~90% of people with cystic fibrosis (CF), but do not work for premature termination codon variants such as W1282X (c.3846G>A). Here we evaluated two gene editing strategies, Adenine Base Editing (ABE) to correct W1282X, and Homology-Independent Targeted Integration (HITI) of a *CFTR* superexon comprising exons 23–27 (SE^23–27^) to enable expression of a *CFTR* mRNA without W1282X. In Flp-In-293 cells stably expressing a CFTR expression minigene bearing W1282X, ABE corrected 24% of W1282X alleles, rescued *CFTR* mRNA from nonsense mediated decay and restored protein expression. However, bystander editing at the adjacent adenine (c.3847A>G), caused an amino acid change (R1283G) that affects CFTR maturation and ablates ion channel activity. In primary human nasal epithelial cells homozygous for W1282X, ABE corrected 27% of alleles, but with a notably lower level of bystander editing, and CFTR channel function was restored to 16% of wild-type levels. Using the HITI approach, correct integration of a SE^23–27^ in intron 22 of the *CFTR* locus in 16HBEge W1282X cells was detected in 5.8% of alleles, resulting in 7.8% of *CFTR* transcripts containing the SE^23–27^ sequence. Analysis of a clonal line homozygous for the HITI-SE^23–27^ produced full-length mature protein and restored CFTR anion channel activity to 10% of wild-type levels, which could be increased three-fold upon treatment with the triple combination of CF modulators. Overall, these data demonstrate two different editing strategies can successfully correct W1282X, the second most common class I variant, with a concomitant restoration of CFTR function.

## Introduction

Cystic Fibrosis (CF) is one of the most common genetic diseases affecting >160 000 people worldwide [1]. It is a recessive disorder caused by variants in the *CFTR* gene, which reduce the expression and/or function of the CFTR anion channel on the surface of secretory epithelial cells [2–4], and leads to obstructions in the lung airways and pancreatic ducts. Of the ~2100 reported variants in the *CFTR* gene, ≥719 have been verified as disease-causing [5] and characterized as to how they affect CFTR protein function [6]. A triple combination of small molecule drugs known as modulators (VX-770, VX-661 and VX-445) is approved for 178 of these variants, which accounts for ~85% of people with CF (pwCF). For some variants, these drugs can restore CFTR activity to approximately 60% of wild-type levels [7], and improve lung function by up to 15 percentage points (FEV_1_ predicted). However, modulator drugs have to be taken continuously as they do not cure the disease [8,9]. Moreover, as these molecules target the CFTR protein, they are ineffective for pwCF who have variants that do not produce any CFTR protein such as the premature termination codon (PTC) variants such as G542X and W1282X, the 2^nd^ and 5^th^ most common CF-causing variants respectively.

PTCs could potentially be treated with read-through molecules that drive incorporation of an amino acid at the PTC site, thereby enabling a full length CFTR protein to be synthesized [10–12]. As PTC-containing transcripts are recognized by the nonsense-mediated decay (NMD) quality control system [13], a second class of drug that can inhibit NMD may be required. It may also be possible to combine these approaches with modulators especially if the CFTR protein generated by readthrough has an alternate amino acid inserted by read-through [11,14], though this combined approach has yet to be evaluated in a clinical trial.

An alternative to small molecule drugs, is the development of gene-editing strategies as a potential treatment for CF [15,16]. We have previously shown efficient correction of the PTC variant W1282X (c.3846G>A), at the DNA level using CRISPR-Cas9 homology directed repair (HDR), and observed ~18% correction of W1282X with concomitant production of wild-type *CFTR* transcripts and expression of functional CFTR protein [17]. However, for every *CFTR* gene bearing W1282X in the cell population that was corrected, roughly the same number of alleles were disrupted by insertions and deletions (indels) generated by non-homologous end joining (NHEJ).

Here, we evaluated two editing techniques that target the W1282X variant. First, we used adenine base editing (ABE) [18] to specifically deaminate c.3846A and restore the wild-type TGG codon at position 1282 in Flp-In-293 cells containing the expression minigene *CFTR* with the W1282X variant [19], and primary human nasal epithelial (HNE) cells homozygous for W1282X. ABE uses a modified TadA deoxyadenosine deaminase fused to a Cas9 nickase with spacer sequence and PAM site chosen such that the target A residue is positioned in an editing window, which typically spans nucleotides 4 to 7 of the spacer. A key advantage of ABE is that the Cas9 nickase causes only a single-strand DNA break as opposed to the double-strand break caused by Cas9 nuclease used for homology directed repair (HDR). Consequently, the level of insertions and deletions (indels) in DNA with ABE is much lower than HDR. A limitation of ABE is that other A residues located in the editing window can also deaminated, a phenomenon known as bystander editing.

The second editing strategy evaluated targeted integration of a superexon (a partial cDNA containing *CFTR* exons 23 to 27 hereinafter referred to as SE^23–27^), into intron 22 of the *CFTR* locus in immortalized isogenic human bronchial epithelial cells (16HBEge) bearing the W1282X variant [20]. Integration and splicing of an SE^23–27^ is attractive as it has the potential to correct any variant occurring in the last 5 exons of *CFTR* (currently W1282X and 92 other CF-causing variants accounting for ~4.5% of people with CF, see Supplementary Material, Table S2). Rather than use a HDR-based strategy, we chose homology-independent targeted integration (HITI), a strategy that strongly favours the integration of the donor in the desired direction [21]. This is achieved by creating a donor that contains the gRNA target site in the reverse complementary orientation to the genomic gRNA recognition site. If the donor integrates in the correct orientation, both sites are destroyed and the donor is locked in the correct orientation, but if the donor integrates in the reverse orientation, two new gRNA targets are created on either side and the donor is typically excised and can be reintegrated in the correct orientation [21]. Our data shows that the two different CRISPR editing systems, both of which have the ability to work in all cell types, and which should retain the normal expression profile of the edited gene, can successfully correct W1282X-CFTR, though further refinements will be required for therapeutic development.

## Results

### Editing with gRNA-A6/NG-ABEmax (NG PAM) efficiently corrects the c.3846G>A (W1282X) variant and significantly increases levels of CFTR mRNA

**Figure 1:**
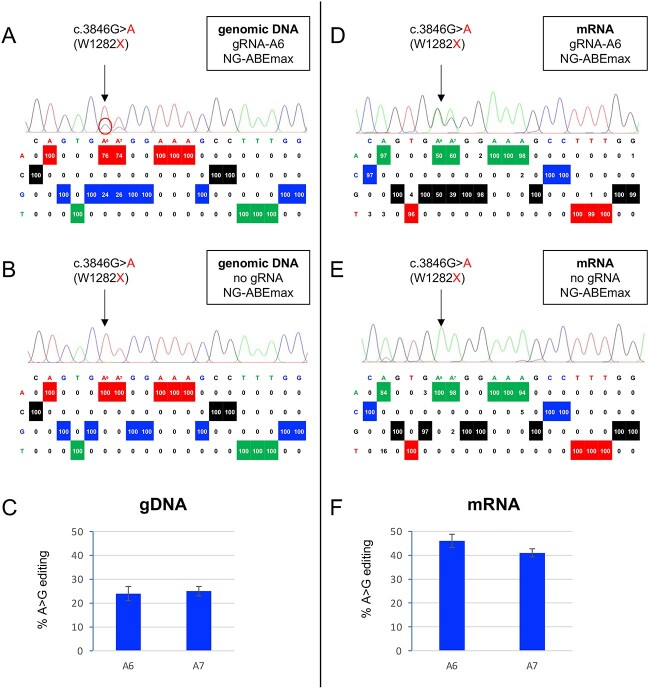
Representative EditR analyses of genomic DNA samples from A) Flp-In-293 W1282X cells transfected with gRNA-A6/NG-ABEmax and B) for NG-ABEmax (no gRNA) transfected cells as a negative control. C) Analysis from four independent DNA experiments showed A>G editing at the target A at position 6 of 24% ± 3% (*n* = 4) and A>G editing at the neighbouring A at position 7 of 25% ± 2% (*n* = 4). Representative EditR analyses of mRNA samples from cells transfected D) with gRNA-A6/NG-ABEmax or E) NG-ABEmax (no gRNA) as a negative control. F) Analysis from three independent mRNA experiments showed A>G editing at the target A at position 6 of 46% ± 2.8% (*n* = 3) and A>G editing at the neighbouring A at position 7 of 41% ± 1.7% (*n* = 3).

The adenine base editor NG-ABEmax [22] can convert adenine (A) to guanine (G) in an editing window that spans nucleotides 4 to 7 of the non-target DNA strand, relative to its NGN PAM site located at nucleotides 21–23. Within this editing window, potentially any adenine can be deaminated, which can result in both precise correction of a target variant, but also unwanted installation of bystander mutations [18]. There is one suitable NGN PAM that positions the target A residue (c.3846G>A) at position 6 (A_6_ gRNA) which could correct the TGA codon to TGG (Supplementary Material, Fig. S1A). We initially evaluated the gRNA-A6/NG-ABEmax combination in the Flp-In-293 W1282X model [19] which contains a single copy of a *CFTR* expression mini-gene containing abridged introns 21, 22, 23, and 24 in the full-length *CFTR* cDNA, and with the c.3846G>A (W1282X) variant in exon 23. Successful A>G base editing at the target A at position 6 was detected in 24% ± 3% of gDNA amplicons (Fig. 1A–C), with no detectable indels. However, the neighbouring A at position 7 (c.3847 of *CFTR* cDNA) was also converted to G at essentially the same level (25% ± 2%). Of note, the closest available NGG PAM placed the target A at position 9 in the non-target strand (see Supplementary Material, Fig. S1B), and as expected, this combination of gRNA with ABE7.10 did not result in any editing of the A in the TGA stop codon (data not shown).

To assess the consequences of these editing events, we first amplified the *CFTR* mRNA by RT-PCR and analysed the sequences by Edit-R which showed that 46% ± 2.8% (*n* = 3) of transcripts had the target A at position c.3846 corrected to G, and 41% ± 1.7% had the adenine at position c.3847 converted to guanine (Fig. 1D–F). The detection of correctly edited transcripts at 1.8-fold higher frequency than the editing of DNA (average 43.5% mRNA vs 24.5% DNA) is likely due to increased stability of the edited RNA transcript that is no longer subject to NMD.

Given that only the A6>G edit would be predicted to rescue the mRNA from NMD, the observation that both edits were enriched in the mRNA at very similar levels suggested the two editing events were strongly linked at the DNA level. To investigate this, and to further characterize the consequences of editing on protein expression and function, we generated 82 clonal cell lines and genotyped them by Sanger sequencing. None of the 82 clones had any detectable indels, but 13 clones containing A to G edits in the target region. Of these 13 clones, the vast majority (77%; *n* = 10) were correctly edited at target A6 but also had the bystander edit at position A7, two clones (15%) were edited at target A6 but also had bystander edits at positions A2 and A7, and just one clone (8% of editing events) was edited at the bystander position A7 alone; none of the clones had the A6 base edited alone. Note, the A2>G bystander editing creates the variant Q1281R and A7>G creates R1283G.

### Editing with gRNA-A6/NG-ABEmax (NG PAM) efficiently restores cell-surface expression of CFTR protein

**Figure 2:**
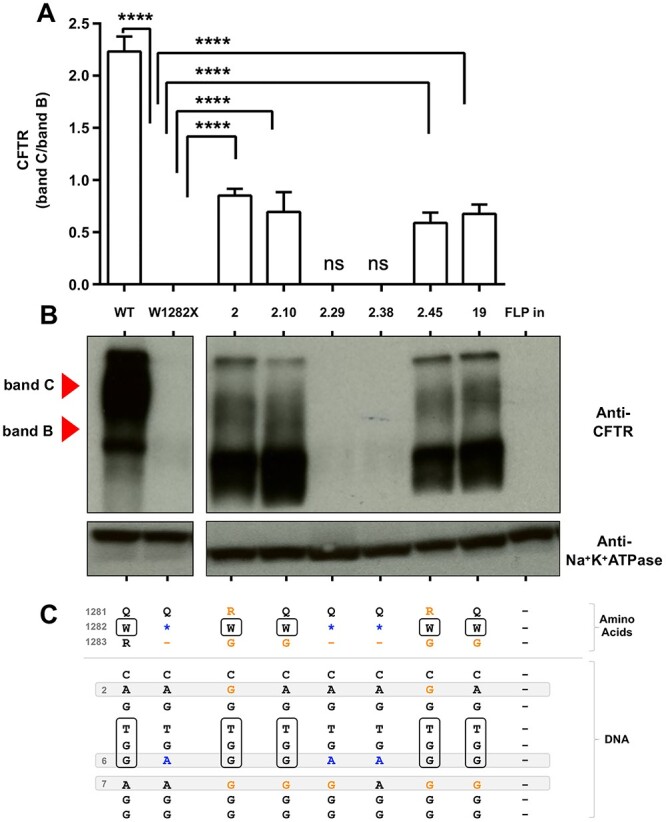
Protein expression by western blot of edited Flp-In-293 W1282X clones. A) Densitometry analysis of western blot data. Bar graph shows quantification of CFTR protein from the immunoblots using ImageJ. Data are represented as the ratio of full-length mature band C/full-length immature band B (mean± SEM, *n* = 3; one-way ANOVA followed by Dunnett’s multiple comparison test, **** p < 0.0001). B) Western blot showing CFTR protein expression in representative clones (isolated from a pool of cells transfected with A6 gRNA and ABEmax-NG) which have no editing (clone 2.38), A7 only edited (clone 2.29), both A6 and A7 edited (clones 2.10 and 19), or A2, A6, and A7 edited (clones 2 and 2.45); Na^+^K^+^ATPase was used as a loading control. C) Predicted protein sequence based on clonal DNA sequence—black = wild-type sequence, blue = uncorrected A6 in PTC codon, orange = bystander edits and corresponding amino acid substitutions, boxed amino acids or nucleotides indicate wild-type sequence at codon 1282.

Cells homozygous for the W1282X variant express virtually no CFTR protein [17,19,20]. To determine if base editing rescued CFTR protein expression and processing, western blot analysis was performed in mock-transfected Flp-In-293 W1282X cells, the pool of edited cells (Fig. 2A), and individual clones isolated from that pool (Fig. 2B–D). As expected, only very low levels of W1282X protein were observed in mock transfected Flp-In-293 W1282X cells (Fig. 2A, left lane and Fig. 2C, left panel–right lane), and in clones 2.29 and 2.38 which did not undergo successful editing at the W1282X target site (Fig. 2C, right panel). In contrast, full-length mature glycosylated (band C) and full-length immature glycosylated (band B) were clearly visible in the pool of Flp-In-293 W1282X edited cells (Fig. 2A, right lane). CFTR protein expression was also substantially restored in individual clones (2.10 and 19) which had the TGA codon repaired and the bystander A7 to G change (which creates the R1283G amino acid change), though the ratio of band C/B was lower than in WT cells (Fig. 2C). This suggests that correction of the W1282X PTC prevents NMD allowing full length protein production, but that installation of the R1283G change causes a defect in protein processing based on the observed low band C/B ratio which is characteristic of class II CF variants such as G85E, F508del or N1303K. Two clones (2 and 2.45) with an additional A2 to G bystander event which creates the Q1281R amino acid change, also had the same protein processing profile as clones 2.10 and 19 (Fig. 2C).

### Adenine base editing of HNE cells results in a partial restoration of CFTR protein function

**Figure 3:**
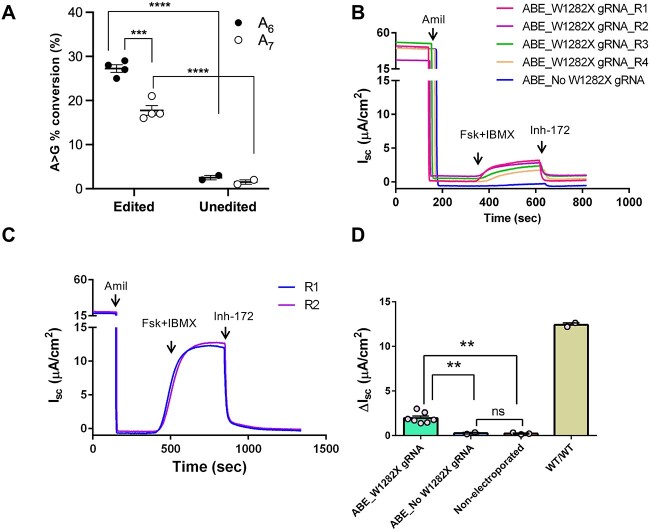
CFTR functional recovery in W1282X homozygous primary nasal cells electroporated with ABE and W1282X gRNA. A) Editing efficiency at A6 and A7 positions. Individual points are from different replicates. Line & error bars denote mean ± SEM. Statistical significance was determined by one-way ANOVA followed by Sidak’s test for multiple comparisons. ****(*p* < 0.0001), ***(*p* < 0.001); B) *I*_sc_ traces of primary HNEs^W1282X/W1282X^ electroporated with A6 gRNA and NG-ABEmax plasmids (four independent transfections plus HNEs transfected with ABE in absence of gRNA). R1, R2, R3, R4 indicate technical replicates; C) *I*_sc_ trace of non-electroporated HNE^WT/WT^; D) Graphical summary of Δ*I*_sc_ data from two biological replicates of W1282X/W1282X HNEs. Δ*I*_sc_ values from WT/WT control are from a single individual; *I*_sc_ trace of non-electroporated HNEs^W1282X/W1282X^ is not shown.

Having established that ABE could correct W1282X and restore full length protein expression (albeit with the R1283G bystander), we decided to evaluate editing in primary human nasal epithelial (HNE) cells. This would enable us to determine if bystander editing varied by cell type, and measure CFTR anion channel function. Primary HNEs homozygous for W1282X were electroporated with A6 gRNA and NG-ABEmax plasmids (*n* = 4), resulting in base editing at both A6 (~27%) and A7 (~18%) (Fig. 3A). Short circuit current was then measured in cells mounted in Ussing chambers, with CFTR function assessed by forskolin activation followed by inhibition with the CFTR-selective inhibitor Inh-172, with Δ*I*_sc_ representing the decrease in current from steady state upon application of Inh-172. W1282X primary HNEs exhibited minimal CFTR function when electroporated with ABE but no gRNA (Fig. 3B, Δ*I*_sc_ = 0.36 μA/cm^2^) compared to CFTR function in untreated WT/WT control (Fig. 3C, Δ*I*_sc_ = 12.4 ± 0.2 μA/cm^2^). However, W1282X primary HNEs electroporated with ABE and A_6_ gRNA resulted in increased CFTR function in all four technical replicates (Fig. 3B, Δ*I*_sc_ = 1.93 ± 0.4 μA/cm^2^). Similar levels of CFTR functional recovery with ABE and A_6_ gRNA were observed in primary HNEs from a second unrelated W1282X homozygous individual, and collectively represented restoration of function at about 16% of WT (Fig. 3D, *n* = 2).

### The CFTR variant R1283G generated as result of bystander effect of W1282X base editing is responsive to VX-445-VX-661-VX-770 combination therapy

**Figure 4:**
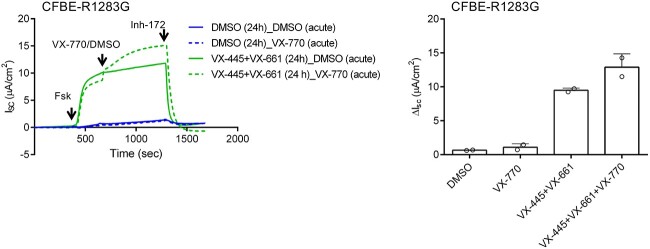
VX-445-VX-661-VX-770 combination therapy partially restores CFTR function in CFBE cells stably expressing R1283G variant. A) Representative short-circuit current (*I*_sc_) recordings in CFBE41o- cells stably expressing R1283G variant. Cell were treated with either DMSO (0.06%) or combination of two correctors VX-445 and VX-661 (3 μM each) for 24 h. VX-770 (10 μM) was added acutely at the time of CFTR function measurement for which cells were mounted on Ussing Chambers. CFTR function was measured under the voltage clamp mode using asymmetric buffer. After the baseline I_sc_ stabilized, forskolin (10 μM, basolateral), VX-770 (10 μM, apical) and CFTR_inh_-172 (Inh-172; 10 μM, apical) were sequentially added at the indicated times. Individual I_sc_ recordings were acquired with Acquire and Analyze software (Physiologic Instruments) and plotted using GraphPad Prism 7.01 software. B) CFTR-specific function defined as the difference (ΔI_sc_) between the sustained phase of the I_sc_ response after stimulation with forskolin followed by Ivacaftor or DMSO as indicated and the baseline achieved after adding CFTR_inh_-172 (mean ± SD). Two different clones of R1283G were tested. Open circle represents mean of two technical replicates from each clone.

To directly test the effect of the R1283G variant on chloride channel activity of CFTR and response to CFTR modulators, we used the CFBE41o- Flp-In cell line which has been used to characterize the function of more than 40 different heterologously expressed CFTR variants [23]. As shown in Fig. 4, the R1283G CFTR variant has negligible levels of chloride channel activity (ΔI_sc_ = 0.7 ± 0.1 μA/cm^2^) which corresponds to less than 0.6% of WT CFTR function observed in Cystic Fibrosis Bronchial Epithelial (CFBE) cells. This observation is consistent with the listing of the c3847A>G variant in the SickKids CFTR database (http://www.genet.sickkids.on.ca) as likely to be a CF-causing variant. Acute treatment with the CFTR potentiator, VX-770 failed to activate R1283G-CFTR function (ΔI_sc_ = 1.1 ± 0.5 μA/cm^2^), but treatment with VX-770 and two CFTR correctors, VX-445 and VX-661, resulted in an ~18-fold increase in chloride channel activity (ΔIsc = 12.9 ± 0.5 μA/cm^2^) corresponding to 10.8% of WT CFTR function in CFBE cells (Fig. 4). CFTR function relative to WT generated by R1283G variant was calculated based on its mRNA expression, and a slope function derived from a linear correlation between *CFTR* mRNA levels and CFTR function from 24 different WT CFTR clones, as reported previously [23].

### Homology-Independent Targeted Integration of Superexon^23–27^ (SE^23–27^) into intron 22 of CFTR locus results in expression of wild-type CFTR mRNA

**Figure 5:**
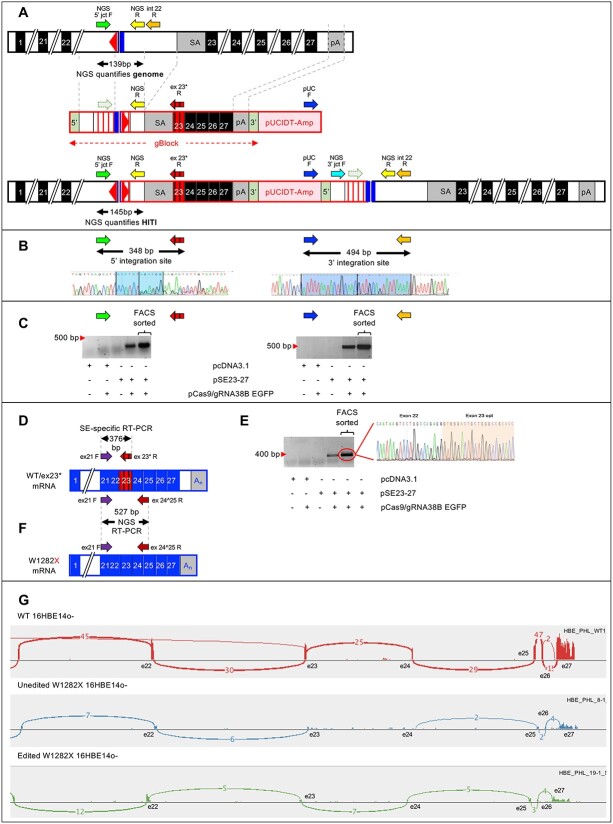
A) Design of superexon 23–27 HITI and PCR strategies to assess integration. The superexon construct starts with 165 bp of *CFTR* intron 22 sequence upstream of the gRNA recognition site, then contains the same gRNA recognition site, but with the PAM and spacer in the reverse complementary orientation, followed by the next 80 bp from intron 22. As detailed in Supplementary Material, Fig. S3, this region contains three PCR primer binding sites (NGS 5′ jct F, NGS R, and int 22 R). To avoid the superexon donor acting as potential template for HDR, the sequence between the NGS 3′ jct F and NGS R primer binding sites has been modified to reduce sequence identity to 66.67% across a 219 bp region; this also prevents the primer NGS 5′ jct F from binding to the superexon construct. The next portion of the superexon is the last 102 bp of intron 22 which comprises the splice acceptor site followed by the 724 bp sequence comprising exons 23–27 and the TAG stop codon. B) Sequence analysis of PCR amplicons to verify correct integration. C) Detection of correct integration at 5′ and 3′ sites in cells enriched for GFP expression. D) Cartoon representation of SE-specific RT-PCR to detect splicing of endogenous exon 21 to ex23* of integrated superexon using primers ex21 F and ex23* R. E) RT-PCR shows detection of splicing of endogenous exon 22 to ex23* only in cells transfected with pSE23-27 and pCas9/gRNA38B. Sequence analysis of RT-PCR from FACS sorted cells confirms correct splice junction of Exon22-Exon23*. F) Cartoon representation of NGS RT-PCR to quantify splicing of endogenous exon 21 to ex23* of integrated superexon using primers ex21 F and ex24^25 R in pool of edited cells. G) Representative sashimi plots of RNA extracted from WT 16HBE14o^−^ (top), unedited 16HBEge W1282X (clone 8—middle) and HITI edited 16HBEge W1282X (clone 19—bottom).

The second method we evaluated to correct the W1282X variant involved the targeted integration of a SE^23–27^ into *CFTR* intron 22 using a Cas9/gRNA target site we had used previously [24]. By designing the construct with the splice acceptor site from the 3′ end of intron 22, the coding sequence of exons 23–27, and ~100 bp of the CFTR 3′UTR and poly(A) sequence (see Supplementary Material, Fig. S2), integration at this site should lead to production of a *CFTR* mRNA comprising exons 1–22 from the *CFTR* locus, spliced to exons 23–27 from the integrated superexon, which contains the wild-type tryptophan (W) codon at position 1282. As shown in Fig. 5A, we used an NHEJ approach known as homology-independent targeted integration (HITI) which strongly favours the integration of the superexon in the desired orientation [21].

Correct integration of the SE^23–27^ was only detected in 16HBEge W1282X cells when transfected with plasmids containing the superexon construct (pSE23-27) and Cas9/gRNA38B Fig. 5B. Two PCRs were performed, one to detect 5′ integration events (NGS 5′jct F and ex23* R = 348 bp product), the other to detect 3′ integration events (pUC F and int22 R = 494 bp product). Sequence analysis of the junctions confirmed correct integration (Fig. 5C). Next, we used RT-PCR to detect expression of an mRNA derived from the splicing of exon 22 to the integrated superexon^23–27^ by using a forward primer that recognizes exon 21 and reverse primer that should only bind to codon optimized exon 23 (ex 21 F and ex23* R = 376 bp product; see Fig. 5D). No product was detected in cells transfected with Cas9/gRNA only or pSE23-27 only, but a band of 376 bp was detected in cells co-transfected with both Cas9/gRNA and pSE23-27. Sequence analysis of this band confirmed that it was derived from transcripts made from splicing the endogenous exons 1–22 to SE^23–27^ (Fig. 5E). Having shown correct integration and expression, we then performed additional characterization to quantitate both processes. Primers NGS 5′ jct F and NGS R (Fig. 5A) were used to generate amplicons for NGS analysis from genomic DNA isolated from a population of cells co-transfected with Cas9/gRNA and pSE23-27 that had been sorted by FACS to enrich for cells that express GFP (from the plasmid pSpCas9(BB)-2A-GFP). Sequence analysis revealed 5.8% (2736/46 555 reads) of genomic DNA amplicons from this population of enriched cells contained the superexon sequence. Primers ex21 F and ex24^25 R were used to amplify a 527 bp product from total RNA from the same population of cells (Fig. 5F). Sequence analysis revealed 7.6% (2603/33 983 reads) contained the mRNA derived from the superexon sequence.

We isolated a clonal cell line with an integrated superexon^23–27^ in both *CFTR* alleles (clone 19) and a control line that went through the editing process but did not contain the integrated SE^23–27^ in either allele (clone 8). Analysis of the *CFTR* mRNA profiles from clone 19 cells revealed 74% of transcripts (Supplementary Material, Fig. S3) were derived from the integrated superexon (with modified exon 23 sequence and a normal W1282 codon), suggesting that in some instances, the splicing machinery skips the superexon and splices to the endogenous exon 23 (containing the W1282X variant). We also performed bulk RNA-sequencing to assess CFTR mRNA abundance. WT 16HBE14o- cells were found to have a substantial number of reads mapping to exon/exon junctions in the exon 22–27 region of CFTR (Fig. 5G, top). In contrast, 16HBEge W1282X cells had very few reads mapping to this region, particularly at the exon 23/exon 24 junction (Fig. 5G, middle). In 16HBEge W1282X cells after HITI (clone 19), there were more reads mapping to CFTR exons 22–27 (Fig. 5G, bottom) as compared to the unedited cell line (clone 8). The number of reads aligning to *CFTR* was visualized using the Integrated Genome Viewer (https://software.broadinstitute.org/software/igv/; Supplementary Material, Fig. S4). Coverage across exon 23 in particular is greater in WT 16HBE14o- as compared to 16HBEge W1282X (clone 8), consistent with the expected decay of W1282X-bearing transcript. Cells containing the integrated SE^23–27^ (clone 19) showed a slight increase in coverage, likely due to stabilization of transcript bearing the superexon. Together, these results indicate that superexon integration results in an increased abundance of stable CFTR transcript.

### Protein expression and functional characterization of SE^23–27^ integrated clone by western blot and I_sc_ measurement

**Figure 6:**
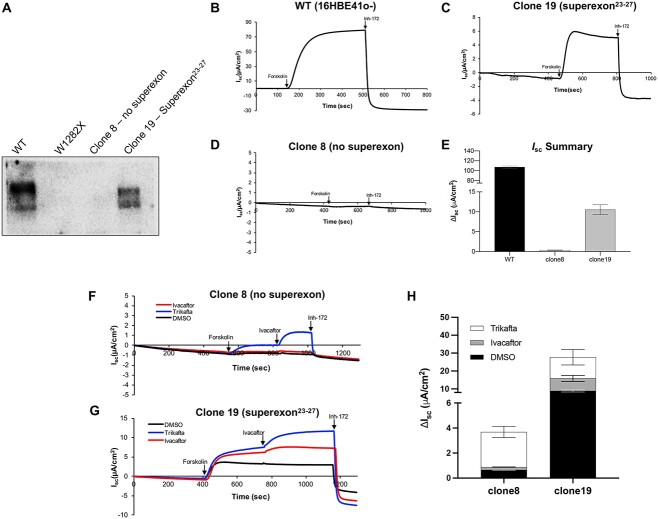
A) Western blot showing CFTR protein expression in the clone 19 with superexon^23–27^ inserted in intron 22. Short circuit current measurements in absence of modulators for B) wild-type cells, C) clone 19 with superexon^23–27^; D) clone 8 without superexon and E) average values. Short circuit current measurements in presence of single (VX-770) or triple (VX-445, VX-661, and VX-770) combination of modulators for F); clone 8 without superexon G) clone 19 with superexon^23–27^; and H) average values.

Western blot analyses of clone 19 with the integrated SE^23–27^ in both *CFTR* alleles showed a *CFTR* expression profile similar to wild-type 16HBE14o- cells (Fig. 6A), although the band C/B ratio was lower than wild-type. To assess CFTR anion function, short circuit current was measured for wild-type cells and both clone 8 and 19, (Fig. 6B–D). While the clone 8 (W1282X, no superexon), did not display a detectable response to forskolin or inh-172, clone 19 (homozygous integration of superexon^23–27^), showed ~10% of CFTR function compared to wild-type 16HBE14o^-^ cells.

Interestingly, when tested with CF modulators, a small increase (<1% to ~4% of WT) was observed in W1282X cells (clone 8) with the triple combination of VX-770, VX-445, and VX-661. In contrast, a more substantial 3-fold increase was observed in the superexon-containing cell line (clone 19) in the presence of VX-770, VX-445, and VX-661; a smaller response was seen with VX-770 alone.

## Discussion

The *CFTR* variant W1282X is the second most common PTC affecting people with CF, with >1700 alleles reported in the CFTR2.org database, and cannot be treated with existing small-molecule drugs. We have previously shown high efficiency correction of this variant by CRISPR-mediated homology directed repair, but for every W1282X allele in a pool of cells that is corrected, on average, an indel is generated in another W1282X allele in a population of cells [17]. Here, we have explored two alternative methods to edit W1282X. First, adenine base editing (ABE) which should restore the TGA (opal stop codon) to the wild-type TGG (tryptophan), and second, homology-independent targeted integration (HITI) of a SE^23–-27^ designed to splice with the endogenous exons 1–22 generating an mRNA encoding wild-type CFTR protein.

Using ABE, we could efficiently correct the W1282X PTC in Flp-In-293 cells, though in most cases, a bystander edit at the adjacent codon (R1283G) in genomic DNA was detected. Editing also resulted in the production of *CFTR* transcripts which evade NMD, which occurs at very high levels for W1282X-containing mRNA [19,25,26]. Base edited cells also showed a substantial rescue of CFTR protein expression, though the band C/B ratio was lower than observed in both wild-type cells and in clonal lines where we corrected W1282X by HDR [17]. The lower band C/B ratio is most likely a consequence of the bystander R1283G.

Base editing was also very effective in primary HNEs homozygous for W1282X with 27% of alleles corrected, and had a consistently lower level of bystander edits compared to that seen in the Flp-In-293 W1282X cells. The level of CFTR function as measured by *I*_sc_ was 16% of wild-type levels which equates to a ratio of 0.6% functional recovery per 1.0% editing. This value is lower than observed values in four recent studies which each reported a roughly linear functional recovery:editing relationship with a ratio of roughly 1.0%–1.5% functional recovery per 1.0% editing for a range of *CFTR* variants in different cell types [17,27–30]. However, based on our observation that alleles with the R1283G bystander edit are non-functional, we can assume that the 16% of WT activity is therefore derived from the 9% of edited alleles that do not have the bystander edit, which equates to a ratio of 1.7:1, a value much closer to the levels reported previously.

Other groups have also observed variation in bystander levels in different cell types and using different delivery methods when using the same gRNA. In human intestinal organoids using electroporation of an xCas9-3.7 ABE plasmid, no bystander was reported with repair of W1282X leading to almost wild-type levels of organoid swelling [31]. Of note, in that study only organoids that showed swelling after 60 min (which indicates genetic and functional restoration of CFTR) were selected; the fact that none of those sequenced had the R1283G bystander further supports the idea that this variant is CF-causing. In contrast, on-target (~38% A6>G) and bystander editing (~33% A7>G) appeared to occur at a similar frequency in primary lung cells when electroporated with Cas9-ABE and gRNA as ribonucleotide particles, although CFTR channel activity was restored to ~75% of wild-type levels [32], though this could be due to a proportion of the bystander edits occurring on the other allele (CFTRdele2,3). A third study of base editing of W1282X which used a different gRNA with the TGA target as residue A9 in the spacer sequence (~24% editing, A9>G) and using ABE RA6.3 reported high levels of bystander editing at position A5 (~48% A5>G) installing Q1281R. However, this amino acid change did not impact protein function [32].

We also showed that the triple combination of CFTR modulators could restore ion channel activity of the bystander R1283G variant to ~10% of WT activity raising the possibility, at least in principle, of combining ABE with modulators. Alternatively, the use of the recently described ABE9 which has a narrower editing window of just 1–2 bp may help reduce the bystander editing [33], though it would need to be modified to recognize an NG PAM. Another option to correct W1282X without the introduction of bystander edits or indels could be to use the recently refined Prime Editing strategies to target W1282X [34].

In this study, we also investigated the use of a superexon strategy to restore CFTR function in the 16HBEge W1282X model, which is homozygous for the W1282X variant [20]. Rather than use a large superexon with the full-length CFTR open reading frame and a homology-directed recombination approach described by others [27,30,35], we used a small superexon (SE^23–27^) comprising a splice acceptor site, *CFTR* exons 23–27 (with exon 23 containing the wildtype but codon-optimized amino acid sequence), and a truncated 3′ UTR and poly(A) sequence, and used the homology-independent targeted integration (HITI) approach [21]. HITI should result in the integration of SE^23–27^ into intron 22 in the correct orientation, and generate mRNA comprising the endogenous *CFTR* exons 1–22 spliced to exons 23–27 of the superexon to produce functional CFTR protein, and should also work in all cell types, regardless of their ability to proliferate [21]. The integration of the SE^23–27^ at the intron 22 target site upstream of the 3849 + 10kbC>T variant also means that this approach could correct any of the 92 different CFTR variants downstream of this site, most of which do not respond to modulators, and account for ~4.5% of pwCF.

The overall level of editing by HITI of the superexon was relatively modest (~6%), and only resulted in a relatively small (33%) increase in the proportion of superexon-derived transcripts (~8%) compared to the much larger (92%) increase in editing-derived transcripts observed with ABE. To investigate this further, and to characterize the level of protein expression and functional restoration, we isolated a clonal line (clone 19) homozygous for the integrated superexon. The observation that only 74% of transcripts contain the wild-type tryptophan residue at position 1282 indicates the splicing to the superexon could be improved; the use of stronger splice acceptor and/or poly(A) sequences may be useful in this regard. This clonal line also showed a restoration of CFTR protein expression, but the band C/B ratio was considerably lower than that in the 16HBE14o- parental line. In addition, the level of CFTR activity measured by *I*_sc_ was only 10% of WT, but a three-fold increase in activity was seen upon modulator treatment.

A number of factors may contribute to these lower-than-expected levels in a clonal line where all alleles contain the integrated SE^23–27^, and where the majority of the *CFTR* transcripts were derived from the superexon. First, at the mRNA level, while 74% of *CFTR* transcripts are derived from the integrated SE^23–27^, the overall level of such transcripts is most likely very low as *CFTR* transcripts that skip the integrated superexon and splice to the W1282X-containing exon 23 will be subject to NMD which occurs at higher level for W1282X relative to many other CF-causing variants [26]. Also, the W1282X-containing *CFTR* transcripts will be targeted by miR-509-3p as they contain the endogenous 3′ UTR [36], whereas we removed virtually all the 3′ UTR (including the miR-509-3p target) in the SE^23–27^ construct. Second, recent work by Harris and colleagues has demonstrated that even in the case of WT 16HBE14o- cells, clonal selection can result in a wide range of *CFTR* expression levels among the derived clones [37]. This variation among the parental cells could have resulted in lower basal *CFTR* expression in 16HBEge W1282X as compared to WT 16HBE14o-. As a result, it is unlikely that the correction of the W1282X mutation through HITI, which is the subsequent step in the process resulting in clone 19, will be able to attain the same *CFTR* expression levels observed in the parental WT 16HBE14o- cells. At the protein level, both the overall level of *CFTR* expression and the ratio of band C/B are lower in clone 19 than WT. One possible explanation is that the codon optimization pattern we used in exon 23 has a negative impact on protein translation, stability, trafficking and/or function. As shown in Supplementary Material, Fig. S5, the codon optimization pattern we used for exon 23 in our SE^23–27^ construct is quite different to the equivalent region of the TI8 superexon used by Suzuki and colleagues [27] who observed a normal CFTR protein band C/B ratio. However, the codon optimization pattern we used is very similar to “FAST” profile for CFTR amino acids 925–983 which resulted in production of CFTR protein with a band C/B ratio of ~1 when expressed in HEK293T cells [38]. Others have also reported substantial effects of CFTR codon optimized protein [39], and variation between the quality control systems of different cell types their ability to detect folding defects [40] may also contribute to the observed differences. Finally, the observation that modulator treatment resulted in a 3-fold increase in CFTR anion channel activity is consistent with codon optimization having a negative impact on protein function; such a large increase in anion channel activity is not typically observed when cells expressing wild-type CFTR are treated with modulators [41]. Overall, this indicates a number of different factors must be taken into account when further developing *CFTR* superexons. It also suggests that if a PE strategy is used to correct W1282X, then the use of mismatches in the RT template sequence of the pegRNA to boost editing efficiency [42] should be carefully selected to avoid disrupting function.

Although both ABE and HITI yield only modest recovery of CFTR function, it has been previously established that the correlation between CFTR channel function and clinical outcomes is non-linear [43]. Therefore, even incremental increases in function are anticipated to yield a clinically significant improvement. Notably, *in vivo* delivery remains a challenge in the translation of genome editing approaches with further studies required to assess the delivery and efficiency of ABE and superexon integration in CF animal models. Nonetheless, HITI superexon integration approaches have been used successfully in models of other diseases such as retinitis pigmentosa [21,44] and adrenoleukodystrophy [45], both ABE and HITI have been used to correct Duchenne’s muscular dystrophy in mice [46,47], additional studies of ABE for CFTR splicing mutants have recently been described [48], and clinical testing of ABE has commenced for heterozygous familial hypercholesterolaemia [49]. Targeting these approaches to the CF lung will be more challenging, however a growing number of non-viral [50–54] and viral options [55,56] for lung delivery are emerging which may facilitate such investigations and add to our understanding of what level of correction may be required for clinical benefit.

## Material and methods

### Cell culture and transfection

Flp-In-293 cells containing the expression minigene *CFTR* with the W1282X variant (Flp-In-293-W1282X-EMG_ai21-ai24 cells) [19,57] were maintained in Dulbecco’s Modified Eagle’s *Medium with 10% FBS,* 1% glutamine, 1% penicillin/streptomycin in the presence of 100 μg/ml Hygromycin. 16HBEge W1282X cells [20], and 16HBE14o- cells [58] were maintained in Eagle’s *Medium with 10% FBS,* 1% glutamine, and 1% penicillin/streptomycin. Cells were transfected with 1 μg DNA and 2 μl Lipofectamine 3000 (Invitrogen) with a 1:1 ratio of gRNA and ABE plasmids, or gRNA/Cas9 and superexon plasmids (HITI) for 2.5x10^5^ cells.

### Design and synthesis of superexon plasmid

Details of the design of the superexon are shown in Fig. 5A and Supplementary Material, Fig. S2. The final DNA sequence was synthesized as a gBlock™ (IDT) and cloned into the pJET1.2/blunt cloning vector (CloneJET PCR Cloning Kit, Thermo Scientific). Fifty-three bp of the 156 bp in exon 23 were changed using the codon optimization tool http://www.jcat.de/ to allow the design of a primer (EX23*R) that only binds exon 23 from superexon to facilitate detection of integration. As shown below, these nucleotide changes also enabled quantification of *CFTR* transcripts derived from splicing to the integrated superexon.

### DNA and RNA extraction and sequencing

16HBE and Flp-In-293 cells were removed from plates and split into two tubes, one for DNA extraction (DNeasy Blood and Tissue Kit, Qiagen), the other for RNA extraction (NucleoSpin® RNA Kit, Macherey-Nagel). For primary HNEs, genomic DNA was collected directly from snapwell filters after short circuit measurements. Reverse transcription was performed using 1 μg of DNase-free RNA (RevertAid H Minus First Strand cDNA Synthesis Kit, ThermoFisher). PCR was performed on gDNA or cDNA (GoTaq® Flexi DNA Polymerase, Promega). Primers are shown in Supplementary Material, Table S1. Amplicons were analysed by Sanger (Eurofins) or deep sequencing (Amplicon-EZ, GENEWIZ). Note that, for deep sequencing, amplicons from the integrated superexon were 145 bp, or 139 bp from the unmodified target region.

### DNA data analysis for editing efficiencies

Base editing was analysed using EditR software [59], indels and knock-in efficiency using ICE (**I**nference of **C**RISPR Edits, Synthego), and deep sequencing raw data using CLC Genomics Workbench software (Qiagen).

### Fluorescence-activated cell sorting (FACS) and cell enrichment

16HBE cells expressing GFP (via the pSpCas9(BB)-2A-GFP plasmid) were quantified and sorted using a BD FACS AriaTM Fusion cell sorter to enrich for cells that were successfully transfected and expressing GFP, then passed through a 70 μm filter prior to seeding as single cells in a 96 well plate.

### Western blotting

Western blots were performed using an anti-CFTR primary antibody (CFFT#596) at 1:3000 to 1:5000 as previously described [17,19].

### Assessment of CFTR channel function in 16HBEge W1282X cells

CFTR channel function in 16HBEge W1282X cells was quantified as previously described [19]. Briefly, cells were grown on snapwell filters for six days then mounted in Ussing chambers to allow for measurement of *I*_sc_. Prior to CFTR activation by forskolin, TMEM16A was inhibited by addition of DIDS. One day prior to performing *I*_sc_ measurements, cells were treated with either corrector compounds (3 μM VX-661 and 3 μM VX-445, Selleckchem) or DMSO applied to the apical media. The potentiator compound VX-770 (10 μM, Selleckchem) or DMSO, was added acutely following the addition of forskolin, but prior to addition of Inh-172.

### Electroporation of primary nasal epithelial cells

Electroporation was performed on primary human nasal epithelial (HNE) cells collected from a W1282X homozygous individual. This study was approved by the Institutional Review Board at Johns Hopkins Medicine, Baltimore, MD (IRB00116966). HNE cells were conditionally reprogrammed using Y-27632 2HCL (Selleckchem) and expanded on irradiated 3 T3 mouse fibroblast as described previously [19,25]. Briefly, 1.5x10^5^ undifferentiated passage 4 cells were electroporated with plasmids at a concentration of 1 μg/μl (base editor plasmid and sgRNA plasmid at a 1:1 molar ratio) using the Neon Transfection System (Invitrogen) with 10 μl Neon tips in buffer R at 1400 V with two 20 ms pulses. Water was used in the no sgRNA control, and pcDNA3-EGFP used for GFP control. Cells were then plated on collagen-coated snapwell filters (Costar) and placed in a six well plate seeded with irradiated 3T3 mouse fibroblasts. Five days post electroporation, filters were moved to fresh wells without irradiated 3T3s. Ten days post-electroporation, cells were moved to differentiation media (PneumaCult ALI-medium; StemCell). Next day apical media was removed, starting air liquid interface (ALI) culture until 20 days post-electroporation at which point short circuit measurements were taken, as previously described [19].

### Generation of cystic fibrosis bronchial epithelial cells stably expressing R1283G and Isc measurements

A CFBE41o- cell line stably expressing the R1283G variant was generated and cultured as previously described [23]. Short-circuit current (*I*_sc_) recordings were made in cells treated with either DMSO (0.06%) or combination of two correctors VX-445 and VX-661 (3 μM each) for 24 h. The potentiator VX-770 (10 μM) was added acutely at the time of CFTR function measurement for which cells were mounted on Ussing Chambers. CFTR function was measured under the voltage clamp mode using asymmetric buffer. After the baseline *I*_sc_ stabilized, forskolin (10 μM, basolateral), VX-770 (10 μM, apical) and CFTR_inh_-172 (Inh-172; 10 μM, apical) were sequentially added at the indicated times. Individual *I*_sc_ recordings were acquired with Acquire and Analyze software (Physiologic Instruments) and plotted using GraphPad Prism 7.01 software. CFTR function relative to WT generated by R1283G variant was calculated based on its mRNA expression, and a slope function derived from a linear correlation between CFTR mRNA levels and CFTR function from 24 different WT CFTR clones, as reported previously [23].

### RNA-sequencing and sashimi analysis

RNA-sequencing was performed at Johns Hopkins Genomics, as previously described [19]. Briefly, RNA was extracted from WT 16HBE14o-, unedited 16HBEge W1282X, and edited 16HBEge W1282X cells. An input of 1.0 μg of RNA was used for library preparation. Five million paired end reads were obtained and aligned to the reference genome (hg19) using STAR v2.7.10a [60]. Sashimi plots were generated using the Integrative Genomics Viewer v2.5.0 [61].

### Statistical analyses

An F-test was performed in Excel to determine if variances of the two population were equal. If they were not, a two-sample t-test was performed. For studies in primary HNEs, ANOVA statistical analysis was performed in GraphPad Prism.

## Supplementary Material

All_Supplemental_figures_and_Tables_FINAL_ddad143

## Data Availability

The authors confirm that the data supporting the findings of this study are available within the article and/or its supplementary materials.
