# Supplementary material for: Use of adenine base editing and homology-independent targeted integration strategies to correct the cystic fibrosis causing variant, W1282X
Source: Hum Mol Genet. 2023 Aug 31;32(23):3237–48. doi: 10.1093/hmg/ddad143 (PMC10656707; doi:10.1093/hmg/ddad143)
Supplement: All_Supplemental_figures_and_Tables_FINAL_ddad143 [file all_supplemental_figures_and_tables_final_ddad143.pdf]

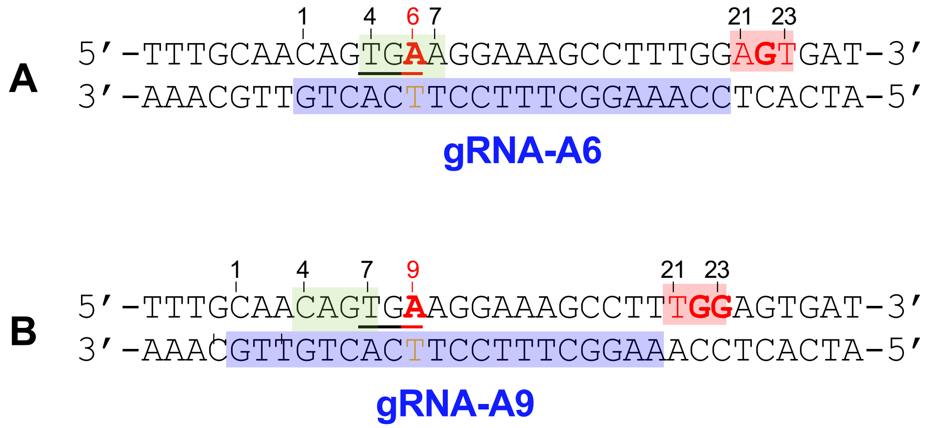

Figure S1: Schematic representation of PAM (red) and spacer (blue) sequence close to c.3846G>A / W1282X, that could be used with **A**) NG-ABEmax (gRNA-A6) and **B**) ABE7.10 (gRNA-A9). The predicted editing window for each gRNA is highlighted in green.

```
                      NGS 5' jct F ---------->
CFTR    gttgaatcattcagtgggtataagcagca...tattctcaatactatgtttcattaataattaatagagatatatgaacacataaaagattcaattataatcaccttgtggatctaaa
              |||||||||||||||||||||||...||||.||.|||.|||.|||.||.|||||.||.||||.|||.|.|.|||.|||.||.|||.|||.||.||||.|.|||.|||.|||.
SE23-27 cagctctcattcagtgggtataagcagcag atattgtccatagtatctttgatgaataagtactagacatagacgtacagatagaacattgaatcatgatcagcatgtcgatgtaac

CFTR    tttcagttgacttgtcatcttgatttctggagaccacaaggtaatgaaaaataattacaagagtcttccatctgttgcagtattaaaatggcgagtaagacaccctgaaaggaaatgt
        ||.|||.|||.|||.|||.||.|||.||||.|||.||.||.||||.||||....|||..|..|......||..|.|||.|.|.||.|||.|||.||.||.||.|.|.||.|||.|.|.
SE23-27 ttgcagatgagttgccatgttcattgctggcgacgacgagctaatcaaaacattttaatactgcaacagatgggatgctgaactagaatcgcGTGTGAGTCAGCGTCAAGGGAGAGGC

                    <---------------- NGS R                                                          <------------ int 22 R
CFTR    tctattcatggtacaatgcaattacagctagcaccaaattcaacactgtttaactttcaacatattattttgatttatcttgatccaacattctcagggaggaggtgcattgaagtta
        ||||.||.|.|||||||||||||||||||||||||...................................................................................
SE23-27 TCTACTCGTCGTACAATGCAATTACAGCTAGCACC-----------------------------------------------------------------------------------

CFTR    ttagaaaacactgacttaagtgtgcaacaaggtttgaatgaataagtgaaaatcttccactggtgacaggataaaatattccaatggttttattgaagtacaatactgaattatgtt
        ....................................................................................................................
SE23-27 ----------------------------------------------------------------------ataaaatattccaatggttttattgaagtacaatactgaattatgtt

                KpnI
CFTR    tatggcatggtacctatatgtcacagaagtgatcccatcacttttaccttatagGTGGGCCTCTTGGGAAGAACTGGATCAGGGAAGAGTACTTTGTTATCAGCTTTTTGAGACTAC
        ||||||||||||||||||||||||||||||||||||||||||||||||||||||||||||||||||||||||||||||||||||||||||||||||||||
SE23-27 tatggcatggtacctatatgtcacagaagtgatcccatcacttttaccttatagGTGGGACTGCTGGGCCGCACCGGCAGCGGCAAGAGCACCCTGCTGAGCGCCTTCCTGCGCCTGC

                                                                      TGA in genome (W128X)
CFTR    TGAACACTGAAGGAGAAATCCAGATCGATGGTGTGTCTTGGGATTCAATAACTTTGCAACAGTGGAGGAAAGCCTTTGGAGTGATACCACAGAAAGTATTTATTTTTTCTGGAACATT
        ||||.||.||.||.||.||.|||||.||.||.|||...|||||....||.||..||||.||||||.|.||.|||||.||.|||||.||.|||||||||||||||||||||||||||||
SE23-27 TGAATACCGAGGGCGAGATTCAGATTGACGGCGTGAGCTGGGACAGCATCACCCTGCAGCAGTGGCGCAAGGCCTTCGGCGTGATCCCCCAGAAAGTATTTATTTTTTCTGGAACATT
                      <------------ ex 23*  R

                                                                                                     HindIII
CFTR    TAGAAAAAACTTGGATCCCTATGAACAGTGGAGTGATCAAGAAATATGGAAAGTTGCAGATGAGGTTGGGCTCAGATCTGTGATAGAACAGTTTCCTGGGAAGCTTGACTTTGTCCTT
        ||||||||||||||||||||||||||||||||||||||||||||||||||||||||||||||||||||||||||||||||||||||||||||||||||||||||||||||||||||||
SE23-27 TAGAAAAAACTTGGATCCCTATGAACAGTGGAGTGATCAAGAAATATGGAAAGTTGCAGATGAGGTTGGGCTCAGATCTGTGATAGAACAGTTTCCTGGGAAGCTTGACTTTGTCCTT

CFTR    GTGGATGGGGGCTGTGTCCTAAGCCATGGCCACAAGCAGTTGATGTGCTTGGCTAGATCTGTTCTCAGTAAGGCGAAGATCTTGCTGCTTGATGAACCCAGTGCTCATTTGGATCCAG
        |||||||||||||||||||||||||||||||||||||||||||||||||||||||||||||||||||||||||||||||||||||||||||||||||||||||||||||||||||||
SE23-27 GTGGATGGGGGCTGTGTCCTAAGCCATGGCCACAAGCAGTTGATGTGCTTGGCTAGATCTGTTCTCAGTAAGGCGAAGATCTTGCTGCTTGATGAACCCAGTGCTCATTTGGATCCAG

CFTR    TAACATACCAAATAATTAGAAGAACTCTAAAACAAGCATTTGCTGATTGCACAGTAATTCTCTGTGAACACAGGATAGAAGCAATGCTGGAATGCCAACAATTTTTGGTCATAGAAGA
        ||||||||||||||||||||||||||||||||||||||||||||||||||||||||||||||||||||||||||||||||||||||||||||||||||||||||||||||||||||||
SE23-27 TAACATACCAAATAATTAGAAGAACTCTAAAACAAGCATTTGCTGATTGCACAGTAATTCTCTGTGAACACAGGATAGAAGCAATGCTGGAATGCCAACAATTTTTGGTCATAGAAGA

CFTR    GAACAAAGTGCGGCAGTACGATTCCATCCAGAAACTGCTGAACGAGAGGAGCCTCTTCCGGCAAGCCATCAGCCCCTCCGACAGGGTGAAGCTCTTTCCCCACCGGAACTCAAGCAAG
        ||||||||||||||||||||||||||||||||||||||||||||||||||||||||||||||||||||||||||||||||||||||||||||||||||||||||||||||||||||||
SE23-27 GAACAAAGTGCGGCAGTACGATTCCATCCAGAAACTGCTGAACGAGAGGAGCCTCTTCCGGCAAGCCATCAGCCCCTCCGACAGGGTGAAGCTCTTTCCCCACCGGAACTCAAGCAAG

CFTR    TGCAAGTCTAAGCCCCAGATTGCTGCTCTGAAAGAGGAGACAGAAGAAGAGGTGCAAGATACAAGGCTTTAG - 1,457bp - ctgccttctcaactccaaactgactcttaagaa
        ||||||||||||||||||||||||||||||||||||||||||||||||||||||||||||||||||||||||.............|||||||||||||||||||||||||||||||||
SE23-27 TGCAAGTCTAAGCCCCAGATTGCTGCTCTGAAAGAGGAGACAGAAGAAGAGGTGCAAGATACAAGGCTTTAG-------------CTGCCTTCTCAACTCCAAACTGACTCTTAAGAA

CFTR    gactgcattatatttattactgtaagaaaatatcacttgtcaataaaatccatacatttgtgtgaaacttgttgttttcagatgcgttcacttgtcatgtttcacgt
        |||||||||||||||||||||||||||||||||||||||||||||||||||||||||||||||||||||..|.|||..||||||||||||||||||||.|||......
SE23-27 GACTGCATTATATTTATTACTGTAAGAAAATATCACTTGTCAATAAAATCCATACATTTGTGTGAAACTGCGCTGTGCTCAGATGCGTTCACTTGTCACGTTCTCGAG
```

Figure S2 – Annotated DNA sequence of gBlock used to make Superexon$^{23-27}$ construct aligned to CFTR genomic sequence. CFTR exons in upper case (Black exons 23, 25 and 27; Grey exons 24 and 26), intron 22 in lower case (grey). Nucleotides highlighted in red in the region before NGS R binding site were introduced to decrease sequence identity to avoid the superexon acting as potential target for HDR. Nucleotides highlighted that are located after the integration site (3 bases upstream of PAM sequence that is underlined) were also used to differentiate between the edited and wild-type sequence in the NGS experiment. Spacer sequence is shown in blue.

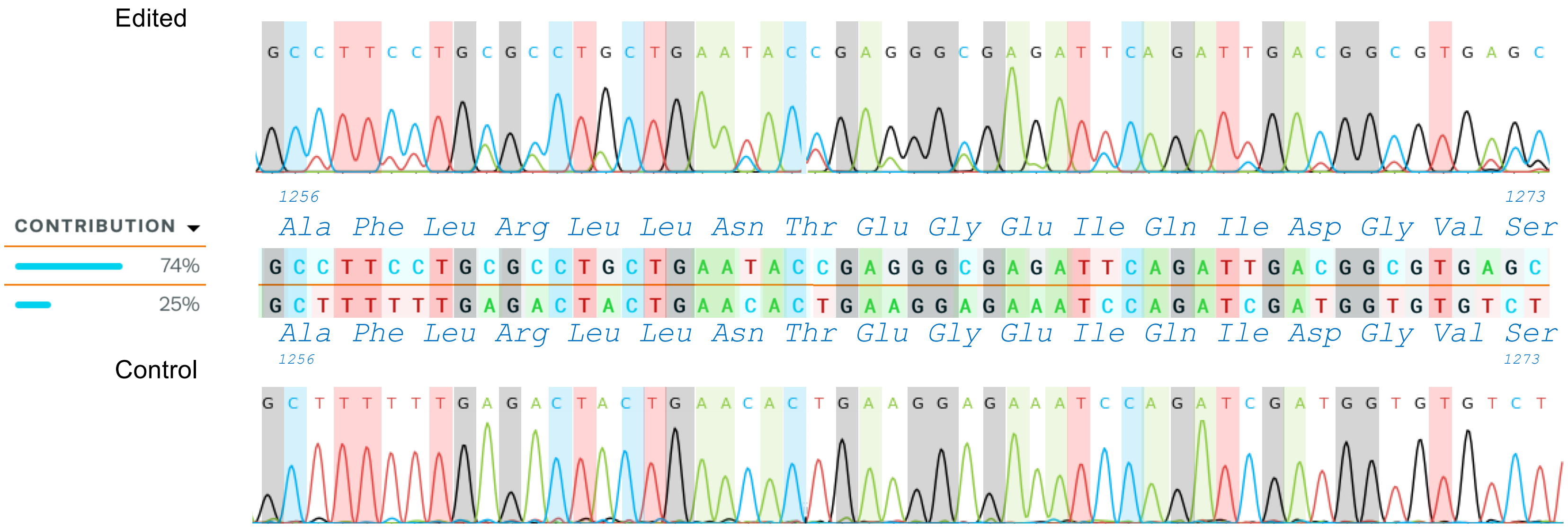

Figure S3. ICE analysis of part of the RT-PCR amplicon from superexon$^{23\text{-}27}$ clonal line 19 as the "edited" sample and an RT-PCR amplicon from unedited cells as "control".

Sequences shown correspond to part of exon 23. ICE analysis shows 74% of sequences from edited sample are from superexon, 25% are from control sample.

Sequences identical in both edited and control are shaded.

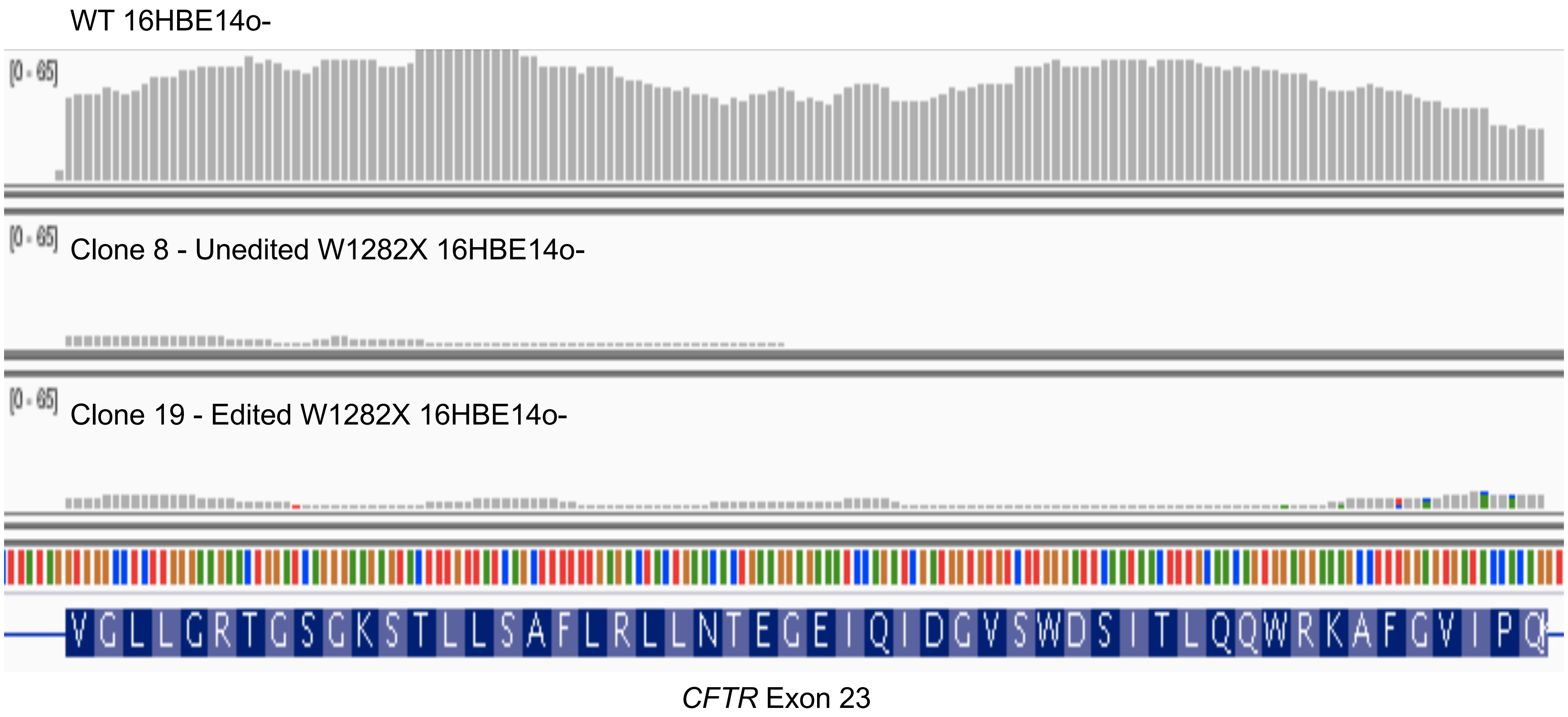

Figure S4: Tracks from IGV showing coverage across the *CFTR* exon 23 reference sequence.

Figure S5.

A-C: Codon optimisation graphs generate from published exon 23 sequences using using https://gcua.schoedl.de

D-F: Codon optimisation graphs generate from published CFTR amino acid 525-593 sequences using using https://gcua.schoedl.de

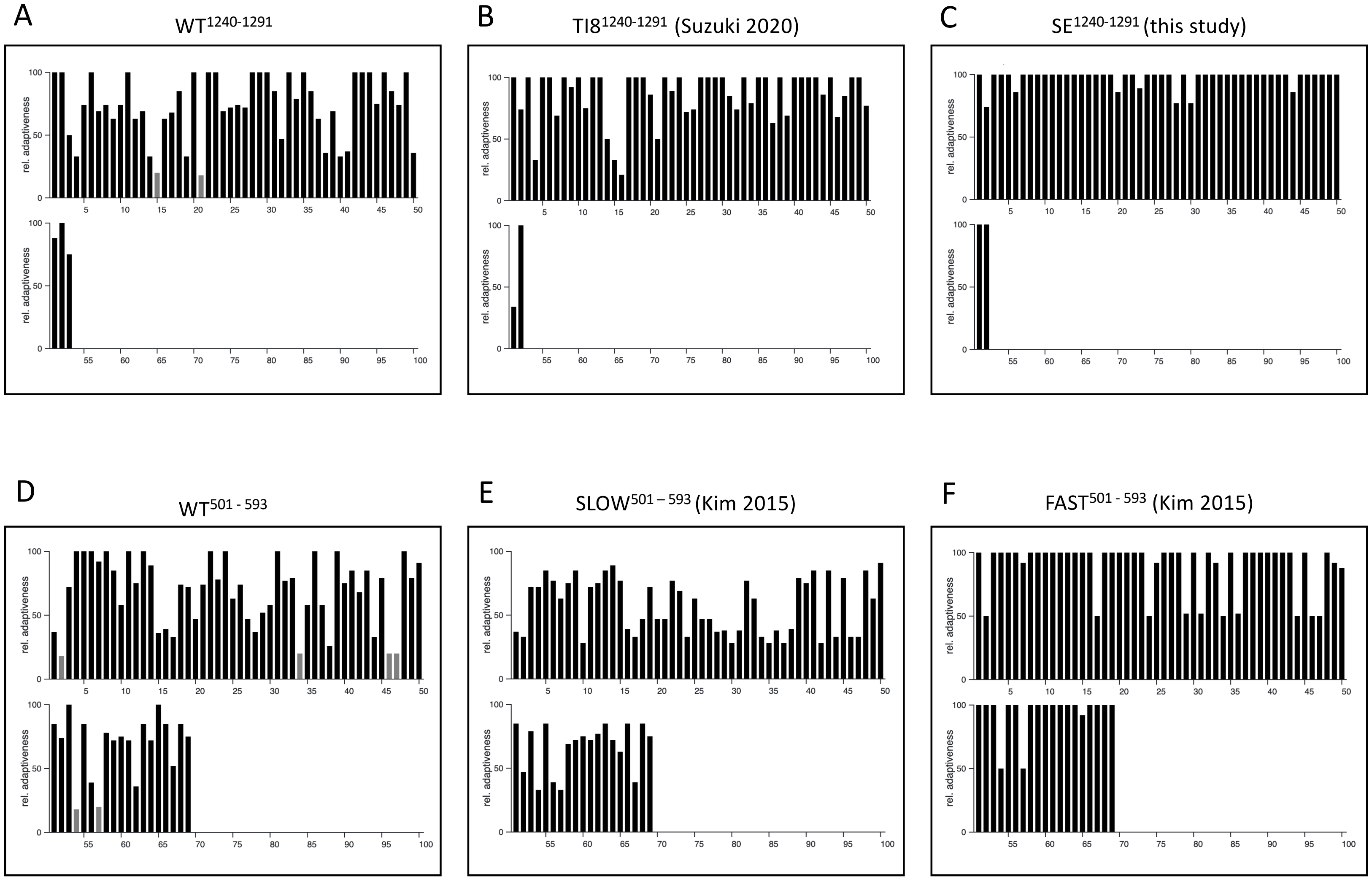

Name	sequence 5' → 3'	Description
NGS F	TCATCTTGATTTCTGGAGACCACA	5’ junction specific for integration
EX23* R	CTCACGCCGTCAATCTGAATCTC	
NGS F	TCATCTTGATTTCTGGAGACCACA	NGS primers
NGS R	GGTGCTAGCTGTAATTGCATTGT	
pBB F	GCGATTAAGTTGGGTAACGCCAG	3’ junction specific for integration
in22 R	ACTTCAATGCACCTCCTCCCTG	
EX21 F	ACTCCAGCATAGATGTGGATAGC	RT-PCR primers
J24-25 R	CCCAACCTCATCTGCAACTTTCC	
GAPDH FW	ACCCACTCCTCCACCTTTGA	RT-PCR controls
GAPDH REV	CTGTTGCTGTAGCCAAATTCGT	
M13legacy20F	tgtaaaacgacggccagtATTGAAGTACAATACTGAATTATG	Amplicon sequencing in HNEs
M13legacy20R	caggaaacagctatgaccGAGTACAAGTATCAAATAGCAG	

Table S1. List of primers.

Table S2. List of 92 CFTR variants downstream of the intron 22 Cas9/gRNA target site. All data from CFTR2.org 7 April 2023 release (The Clinical and Functional TRanslation of CFTR (CFTR2); available at https://cftr2.org).

Notes

(1) Represents the allele frequency within the CFTR2 database.This is subject to regional and ethnic variability of variant distribution and may differ from the worldwide frequency.
(2) frequency expressed as %
(3) CF = CF-causing, VCC = varying clinical consequence
(4) dele22,23 refers to the legacy exon nomenclature

intron / EXON	Variant cDNA name (ordered 5' to 3')	Variant protein name	Variant legacy name	# alleles in CFTR2	Allele frequency (1,2)	CF/VCC (3)
in 22	c.3718-2477C>T	p.?	3849+10kbC->T	1,158	0.8153%	CF
in 22	c.3718-3T>G	p.?	3850-3T->G	6	0.0042%	CF
in 22	c.3718-1G>A	p.?	3850-1G->A	12	0.0084%	CF
EX 23	c.3719T>G	p.Val1240Gly	V1240G	3	0.0021%	CF
EX 23	c.3724del	p.Leu1242SerfsX17	3856delC	1	0.0007%	CF
EX 23	c.3731G>A	p.Gly1244Glu	G1244E	106	0.0746%	CF
EX 23	c.3737C>T	p.Thr1246Ile	T1246I	23	0.0162%	VCC
EX 23	c.3743C>A | c.3743C>G	p.Ser1248X	S1248X	1	0.0007%	CF
EX 23	c.3744del	p.Lys1250ArgfsX9	3876delA	91	0.0641%	CF
EX 23	c.3745G>A	p.Gly1249Arg	G1249R	7	0.0049%	CF
EX 23	c.3747del	p.Lys1250ArgfsX9	3878delG	9	0.0063%	CF
EX 23	c.3752G>A	p.Ser1251Asn	S1251N	120	0.0845%	CF
EX 23	c.3754dup	p.Thr1252AsnfsX13	3886insA	1	0.0007%	CF
EX 23	c.3758dup	p.Leu1253PhefsX12	3889dupT	1	0.0007%	CF
EX 23	c.3761T>G	p.Leu1254X	L1254X	24	0.0169%	CF
EX 23	c.3763T>C	p.Ser1255Pro	S1255P	10	0.0070%	CF
EX 23	c.3764C>A	p.Ser1255X	S1255X	16	0.0113%	CF
EX 23	c.3767dup	p.Leu1258PhefsX7	3898insC	1	0.0007%	CF
EX 23	c.3773del	p.Leu1258X	3905delT	1	0.0007%	CF
EX 23	c.3773dup	p.Leu1258PhefsX7	3905insT	210	0.1478%	CF
EX 23	c.3796G>T	p.Glu1266X	E1266X	1	0.0007%	CF
EX 23	c.3806T>A	p.Ile1269Asn	I1269N	12	0.0084%	CF
EX 23	c.3808del	p.Asp1270MetfsX8	3940delG	3	0.0021%	CF
EX 23	c.3808G>A	p.Asp1270Asn	D1270N	55	0.0387%	VCC
EX 23	c.3816_3817del	p.Ser1273LeufsX28	3944delGT	1	0.0007%	CF
EX 23	c.3822G>A	p.Trp1274X	W1274X	3	0.0021%	CF
EX 23	c.3827del	p.Ser1276X	3959delC	1	0.0007%	CF
EX 23	c.3829del	p.Ile1277X	3960-3961delA	1	0.0007%	CF
EX 23	c.3838C>T	p.Gln1280X	Q1280X	1	0.0007%	CF
EX 23	c.3841C>T	p.Gln1281X	Q1281X	2	0.0014%	CF
EX 23	c.3846G>A	p.Trp1282X	W1282X	1,726	1.2152%	CF
EX 23	c.3848G>T	p.Arg1283Met	R1283M	7	0.0049%	CF
EX 23	c.3872A>G	p.Gln1291Arg	Q1291R	9	0.0063%	VCC
EX 23	c.3873G>C	p.Gln1291His	Q1291H	30	0.0211%	VCC
in 23	c.3873+1G>A	p.?	4005+1G->A	21	0.0148%	CF
in 23	c.3873+2T>C	p.?	4005+2T->C	15	0.0106%	CF
in 23	c.3874-1G>A	p.?	4006-1G->A	3	0.0021%	CF

Table 2 (continued)

intron / EXON	Variant cDNA name (ordered 5' to 3')	Variant protein name	Variant legacy name	# alleles in CFTR2	Allele frequency (1,2)	CF/VCC (3)
EX 24	c.3876del	p.Val1293TyrfsX35	4006delA	1	0.0007%	CF
EX 24	c.3883_3886del	p.Ile1295PhefsX32	4010del4	12	0.0084%	CF
EX 24	c.3883del	p.Ile1295PhefsX33	4015delA	5	0.0035%	CF
EX 24	c.3889dup	p.Ser1297PhefsX5	4016insT	100	0.0704%	CF
EX 24	c.3891dup	p.Gly1298TrpfsX4	4022insT	5	0.0035%	CF
EX 24	c.3904A>T	p.Lys1302X	K1302X	2	0.0014%	CF
EX 24	c.3908del	p.Asn1303ThrfsX25	4040delA	10	0.0070%	CF
EX 24	c.3909C>G	p.Asn1303Lys	N1303K	2,246	1.5813%	CF
EX 24	c.3917_3918dup	p.Tyr1307ProfsX22	4048insCC	1	0.0007%	CF
EX 24	c.3921T>A	p.Tyr1307X	Y1307X	2	0.0014%	CF
EX 24	c.3925C>T	p.Gln1309X	Q1309X	1	0.0007%	CF
EX 24	c.3929G>A	p.Trp1310X	W1310X	3	0.0021%	CF
EX 24	c.3932_3933delinsAATATG	p.Ser1311LysfsX12	4064-4065delinsAATATG	1	0.0007%	CF
EX 24	c.3937C>T	p.Gln1313X	Q1313X	30	0.0211%	CF
EX 24	c.3947G>A	p.Trp1316X	W1316X	2	0.0014%	CF
EX 24	c.3957del	p.Asp1320MetfsX8	4089delA	1	0.0007%	CF
in 24	c.3963+1G>A	p.?	4095+1G->A	1	0.0007%	CF
in 24	c.3963+1G>C	p.?	4095+1G->C	1	0.0007%	CF
in 24	c.3964-78_4242+577del	p.?	CFTRdele22,23 (4)	40	0.0282%	CF
EX 25	c.3971T>C	p.Leu1324Pro	L1324P	4	0.0028%	CF
EX 25	c.3976del	p.Ser1326LeufsX2	4108delT	2	0.0014%	CF
EX 25	c.3988C>T	p.Gln1330X	Q1330X	3	0.0021%	CF
EX 25	c.4004T>C	p.Leu1335Pro	L1335P	19	0.0134%	CF
EX 25	c.4028dup	p.Cys1344LeufsX15	4160insG	2	0.0014%	CF
EX 25	c.4033_4034del	p.Val1345ProfsX13	4165delGT	1	0.0007%	CF
EX 25	c.4035_4038dup	p.Ser1347ProfsX13	p.S1347PfsX13	1	0.0007%	CF
EX 25	c.4036_4042del	p.Leu1346MetfsX6	4168delCTAAGCC	3	0.0021%	CF
EX 25	c.4040_4041del	p.Ser1347ThrfsX11	4172delGC	1	0.0007%	CF
EX 25	c.4046del	p.Gly1349AlafsX5	4177delG	1	0.0007%	CF
EX 25	c.4046G>A	p.Gly1349Asp	G1349D	22	0.0155%	CF
EX 25	c.4051A>T	p.Lys1351X	K1351X	1	0.0007%	CF
EX 25	c.4065_4066del	p.Leu1356GlyfsX2	4197_4198delCT	1	0.0007%	CF
EX 25	c.4071_4073delinsAA	p.Arg1358AsnfsX22	4203TAG->AA	1	0.0007%	CF
EX 25	c.4077_4080delinsAA	p.?	4209TGTT->AA	18	0.0127%	CF
EX 25	c.4086dup	p.Lys1363X	4218insT	5	0.0035%	CF
EX 25	c.4090del	p.Ala1364ArgfsX16	4222delG	1	0.0007%	CF
EX 25	c.4097T>A	p.Ile1366Asn	I1366N	5	0.0035%	CF
EX 25	c.4111G>T	p.Glu1371X	E1371X	19	0.0134%	CF
EX 25	c.4124A>C	p.His1375Pro	H1375P	7	0.0049%	CF
EX 25	c.4127_4131del	p.Leu1376SerfsX8	4259del5	3	0.0021%	CF
in 25	n/a	n/a	n/a	0	n/a	n/a
EX 26	c.4139del	p.Thr1380AsnfsX4	4271delC	1	0.0007%	CF
EX 26	c.4144C>T	p.Gln1382X	Q1382X	5	0.0035%	CF
EX 26	c.4147dup	p.Ile1383AsnfsX3	4279insA	5	0.0035%	CF
EX 26	c.4168C>T	p.Gln1390X	Q1390X	3	0.0021%	CF
EX 26	c.4170del	p.Ala1391HisfsX7	4301delA	1	0.0007%	CF
EX 26	c.4197_4198del	p.Cys1400X	4326delTC	18	0.0127%	CF
EX 26	c.4231C>T	p.Gln1411X	Q1411X	7	0.0049%	CF
EX 26	c.4234C>T	p.Gln1412X	Q1412X	4	0.0028%	CF
in 26	c.4242+1G>A	p.?	4374+1G->A	8	0.0056%	CF
in 26	c.4242+1G>T	p.?	4374+1G->T	16	0.0113%	CF
EX 27	c.4251del	p.Glu1418ArgfsX14	4382delA	65	0.0458%	CF
EX 27	c.4300_4301dup	p.Ser1435GlyfsX14	4428insGA	4	0.0028%	CF
EX 27	c.4364C>G	p.Ser1455X	S1455X	18	0.0127%	VCC
EX 27	c.4426C>T	p.Gln1476X	Q1476X	17	0.0120%	VCC
EX 27	c.4439T>C	p.Leu1480Pro	L1480P	5	0.0035%	VCC
			TOTAL	6,453	4.5432%	
